# Antiviral Peptide-Generative Pre-Trained Transformer (AVP-GPT): A Deep Learning-Powered Model for Antiviral Peptide Design with High-Throughput Discovery and Exceptional Potency

**DOI:** 10.3390/v16111673

**Published:** 2024-10-25

**Authors:** Huajian Zhao, Gengshen Song

**Affiliations:** Beijing Youcare Kechuang Pharmaceutical Technology Co., Ltd., Beijing 100176, China; zhaohuajian@youcareyk.com

**Keywords:** antiviral peptide design, transformer, generative modeling, drug discovery, high throughput

## Abstract

Traditional antiviral peptide (AVP) discovery is a time-consuming and expensive process. This study introduces AVP-GPT, a novel deep learning method utilizing transformer-based language models and multimodal architectures specifically designed for AVP design. AVP-GPT demonstrated exceptional efficiency, generating 10,000 unique peptides and identifying potential AVPs within two days on a GPU system. Pre-trained on a respiratory syncytial virus (RSV) dataset, AVP-GPT successfully adapted to influenza A virus (INFVA) and other respiratory viruses. Compared to state-of-the-art models like LSTM and SVM, AVP-GPT achieved significantly lower perplexity (2.09 vs. 16.13) and higher AUC (0.90 vs. 0.82), indicating superior peptide sequence prediction and AVP classification. AVP-GPT generated a diverse set of peptides with excellent novelty and identified candidates with remarkably higher antiviral success rates than conventional design methods. Notably, AVP-GPT generated novel peptides against RSV and INFVA with exceptional potency, including four peptides exhibiting EC50 values around 0.02 uM—the strongest anti-RSV activity reported to date. These findings highlight AVP-GPT’s potential to revolutionize AVP discovery and development, accelerating the creation of novel antiviral drugs. Future studies could explore the application of AVP-GPT to other viral targets and investigate alternative AVP design strategies.

## 1. Introduction

Peptides have emerged as promising tools in the fight against various infectious diseases, including viral infections [1]. Enfuvirtide, an FDA-approved anti-HIV peptide, serves as a testament to their potential [2]. Human respiratory syncytial virus (RSV) and influenza A virus (INFVA) are two of the major human respiratory pathogens, and numerous researchers have worked to create therapeutic peptides that can fight both viruses. Derived from the HR1 or HR2 domains in the RSV F protein, peptides inhibit RSV fusion by binding to HR2 or HR1 and blocking the formation of the 6-HB core [2]. Peptides derived from sequences of the HA1 and HA2 subunits of INFVA have shown anti-INFVA activity, preventing the HA conformational changes required to carry out the membrane fusion event [3]. It is vitally necessary to discover effective antiviral medications for the prevention and treatment of RSV, INFVA, and other respiratory viruses, as no peptides that effectively stop the spread of RSV, INFVA, and other respiratory viruses have been reported. However, developing effective antiviral peptides traditionally involves a time-consuming and expensive process of library creation and experimental screening.

To address these limitations, machine learning models have gained traction for high-throughput peptide design and antiviral activity identification [4,5]. A recurrent neural network (RNN) model was used for the generation of antibacterial peptides, and 82% of the peptides were predicted to be active antibacterials [4]. State-of-the-art models, support vector machine (SVM) and random forest (RF), were utilized for the screening of antiviral activity based on peptide descriptors and have shown promising results in AVP prediction. [5,6]. However, these models rely on hand-crafted features, which can be time-consuming to extract and may not capture the full complexity of peptide sequences. Notably, transformer-based deep learning models show exceptional promise due to their ability to capture complex relationships within data [7]. Transformers leverage an attention mechanism to learn intricate aspects of input sequences, leading to more comprehensive interpretations compared to other models. While transformers like BERT (Bidirectional Encoder Representations from Transformers) excel in classification tasks, and GPT (Generative Pre-training Transformer) demonstrates exceptional language understanding and generation capabilities [8,9], their application in peptide design remains unexplored.

In this study, we introduce AVP-GPT, a novel method for antiviral peptide design that leverages the power of transformer models. The RSV dataset was used for the pre-training process, and the INFVA and other respiratory virus datasets were employed for the fine-tuning process in this work, since the RSV dataset is larger than the INFVA and other respiratory virus datasets. We compare AVP-GPT’s performance to state-of-the-art methods and showcase its superior efficiency in generating unique peptides and identifying potential antiviral candidates. The different components and strategies within AVP-GPT are designed to enhance prediction accuracy, as confirmed by an ablation study. Additionally, we perform a comparison study and phylogenetic analysis to validate the novelty of peptides generated by AVP-GPT. Notably, AVP-GPT can generate 10,000 distinct peptides and identify potential AVP candidates in less than two days on a GPU system. Finally, we present the results of an in vitro investigation that validates the potent antiviral activity of AVP-GPT-discovered peptides. To the best of our knowledge, this is the first study to successfully design antiviral peptides with exceptional activity using transformer-based architectures.

## 2. Materials and Methods

### 2.1. Datasets

**Figure 1:**
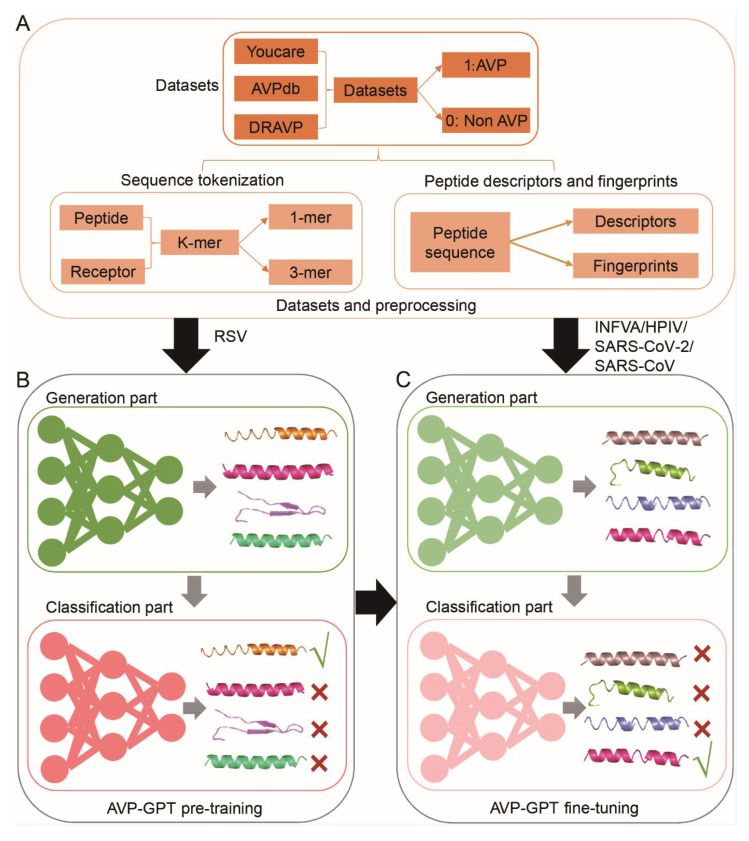
AVP-GPT workflow for AVP creation and identification. (**A**) Data collection and preprocessing: Antiviral sequence data were gathered from our company’s databases (Youcare) and external resources (AVPdb and DRAVP). The data were then converted into a format compatible with AVP-GPT. This involves the following steps: sequence tokenization, peptide descriptor calculation, and fingerprint generation. (**B**) Pre-training: AVP-GPT underwent pre-training using RSV data. This initial training helps the model learn general patterns and underlying principles from known antiviral sequences. (**C**) Fine-tuning: The pre-trained AVP-GPT model was further refined using INFVA, HPIV, SARS-CoV-2, and SARS-CoV data. This fine-tuning step specializes AVP-GPT in producing and identifying antiviral peptides specifically against these respiratory viruses.

We compiled antiviral peptide sequences from our group (named Youcare), AVPdb [10], and DRAVP [11] (Figure 1). The Youcare dataset is a collection of manually designed peptides targeting RSV, distinct from public data available from AVPdb and DRAVP. To ensure data quality and consistency, we removed duplicate sequences from the datasets and converted EC50/IC50 values to binary labels (0: non-AVPs, 1: AVPs). The non-redundant dataset for pre-training AVP-GPT included 412 non-redundant sequences targeting RSV, 47 sequences targeting INFVA, 66 sequences targeting SARS-CoV, 60 sequences targeting SARS-CoV-2, and 19 sequences targeting HPIV. The RSV dataset was the largest, with 61% of peptides having IC50/EC50 values ≥ 10 µM. To maintain a relatively balanced dataset, we set the antiviral activity threshold to 10 µM.

### 2.2. Sequence Tokenization

Following a recent k-mer representation method [12], we tokenized both peptide and receptor sequences into k-mers. For the generation model, we used a k-mer length of 1, while a k-mer length of 3 was used for the classification model (Figure 1). For example, the sequence “NFYDPL” can be tokenized to a sequence of four 3-mers: NFY, FYD, YDP, and DPL. Peptide modifications were considered special tokens and not further split during tokenization (e.g., PEG modification becomes “<M1>”). The maximum input length was set to 97 for the generation model, which only accepts peptide tokens. The classification model, receiving both receptor and peptide tokens, has a maximum input length of 401. A total of 26 tokens were produced for the generation model: A, R, N, D, C, Q, E, G, H, I, L, K, M, F, P, S, T, W, Y, V, <M1>, <M2>, <M3>, <unknown>, <start>, and <pad>. For the classification model, 8509 tokens were generated, including SLT, NIT, TTT, KKL, KLN, LLS, LSK, KLI, SLI, and ILK. The RSV HR1 sequence (NCBI accession FJ614815), the HR sequence of INFVA (PDB code 5VLI), the HPIV receptor sequence (NCBI accession U51116.1), the SARS-CoV receptor sequence (NCBI accession DQ231462.2), and the SARS-CoV-2 receptor sequence (PDB code 7COT_A) were predicted by LearnCoil-VMF [13]. The RSV HR2 reference sequence was NFYDPLVFPSDEFDASISQVNEKINQSLAFIRKSDELLHNVNAGKSTTN [2].

### 2.3. Peptide Descriptors

To capture various physicochemical properties of the peptides, we employed ifeatpro (https://github.com/deeprob/ifeatpro, accessed on 31 January 2024) and peptides (https://github.com/althonos/peptides.py, accessed on 31 January 2024) to calculate several peptide descriptors. These descriptors included the following:

- Amino acid composition (AAC): the overall frequency of each amino acid type present in the peptide sequence [6].
- Dipeptide composition (DPC): the frequency of each unique dipeptide (two consecutive amino acids) combination within the sequence [6].
- CKSAAGP: a descriptor set capturing properties like aliphatic, aromatic, and charge distribution [6].
- Pseudo-amino acid composition (PAAC): a broader representation of the amino acid composition that considers additional features beyond just the amino acid type [14].
- Physicochemical features (PHYC): a set of descriptors that encode various physicochemical properties of the peptide, such as alpha-helical propensity, isoelectric point, and hydrophobicity [6].

Finally, a total of 626 peptide descriptors features were generated. For AVP-GPT, we utilized all 626 features without initial selection. This decision was made because AVP-GPT’s architecture is designed to handle high-dimensional data and potentially learn important relationships between features during the training process. Using a variety of peptide descriptors allows the model to capture a broader range of information about the peptides, potentially leading to improved performance. On the other hand, for SVM and RF models that are more sensitive to feature dimensionality, we performed feature selection using Lasso. By applying Lasso, we identified the most informative features that contribute significantly to model performance in these specific models.

### 2.4. Peptide Fingerprints

In addition to descriptors, we also generated fingerprint representations of the peptides using RDKit (https://www.rdkit.org, accessed on 31 January 2024). This method involves two steps: converting the peptide sequence into a SMILES string and transforming the SMILES string into a fingerprint.

### 2.5. Generation Part of AVP-GPT Architectures

**Figure 2:**
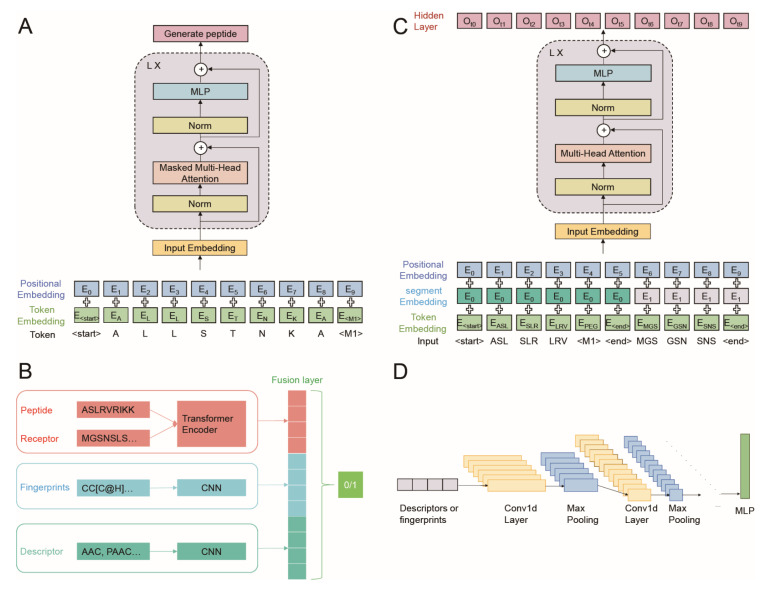
AVP-GPT model architecture with key features. (**A**) Generation part: This part is responsible for creating new peptide sequences. It utilizes a transformer decoder, a deep learning architecture adept at handling sequential data like peptide sequences. Sequence tokens, representing individual amino acids and special characters (e.g., “<start>”, “<M1>”), are fed into the transformer decoder. (**B**) Classification part: This part determines whether a given peptide sequence has antiviral properties. It employs separate 1D CNNs to process two different peptide representations: peptide descriptors and peptide fingerprints. The outputs from these three components (peptide descriptor CNN, peptide fingerprint CNN, and transformer encoder) are concatenated before feeding them into a final layer for binary classification (AVP vs. non-AVP). (**C**) Sequence tokens representing individual amino acids and special characters (e.g., “<start>”, “<M1>”,”<end>”) are fed into the transformer encoder. (**D**) Peptide descriptors and fingerprints are processed by separate 1D CNNs. Note: “<M1>” is a special token used within the sequence that signifies PEG (polyethylene glycol) modification of the peptide.

This part includes the following (Figure 2A):

- Input embedding: set as learnable parameters, positional encodings were added to the input embedding of sequence tokens and modification tokens.
- Masked multi-head attention: this layer allows each output to only pay attention to the past tokens.
- Layer normalization and residual connections: these techniques are applied after each block to improve training stability and gradient flow.
- Peptide generation process, which included the following:
  1. A “<start>” token is fed as input.
  2. The model iteratively predicts the next amino acid and modification (if applicable) based on the current sequence.
  3. The temperature parameter controls the randomness of the generated sequence.
  4. Top-k sampling selects the next token based on the top-k most probable options.
  5. Max_new_tokens defines the maximum length of the generated peptide.
- Loss function: cross-entropy with ignore_index = 0 is used to calculate the loss during training, ignoring padded “<pad>” tokens.

### 2.6. Classification Part of AVP-GPT Architectures

This part utilizes a combination of a transformer encoder and two CNN models to process different data modalities (Figure 2B):

- Transformer encoder: handles peptide sequences and receptor sequences with segment embeddings and positional encodings added to the input embedding (Figure 2C).
- CNN models: two separate CNN models process peptide descriptors and peptide fingerprints.
- Fusion layers: the hidden outputs from the transformer encoder and both CNN models are concatenated in the fusion layers (Figure 2D).
- Binary classification: the final output predicts whether the peptide is an AVP (antiviral) or not.

### 2.7. Improving Robustness

To enhance the robustness of the classification model during training, 50% of the receptor sequences, peptide fingerprints, and peptide descriptors are randomly removed and replaced with padding (“<PAD>” or zero).

### 2.8. AVP-GPT Pre-Training

We pre-trained AVP-GPT on a dataset of RSV peptide sequences. The following is a breakdown of the hyperparameters used for each part of the model:

#### 2.8.1. Generation Part

This part involved the following:

- Transformer decoders: 3.
- Self-attention heads: 3.
- Dropout rate: 0.5.
- Learning rate: 0.0003.
- Output dimension: 26 (number of tokens).
- Maximum generated tokens: 36 (peptide length limit).
- Temperature: 1 (controls randomness).
- Top-k sampling: 24 (considers top 24 probable next tokens). These are used during inference only.

#### 2.8.2. Classification Part

This part involved the following:

- Transformer encoders: 2.
- Self-attention heads: 3.
- Dropout rate: 0.5.
- Output dimension: 64 (for transformer encoders).
- CNN layers (peptide descriptors): 3 layers with kernel size 3, dropout 0.5, and output dim 64.
- CNN layers (peptide fingerprints): 3 layers with kernel size 3, dropout 0.5, and output dim 16.

### 2.9. Training Setup

The RSV data were split into training (60%), validation (20%), and testing (20%) sets. We trained all models using the Nadam optimizer [15] with a batch size of 64 for 500 epochs on an NVIDIA^®^ V100 Tensor Core GPU.

### 2.10. Evaluation Methods

The following evaluation methods were used:

- Perplexity: Used to compare AVP-GPT’s generation performance to an LSTM model, a common approach for peptide generation. Lower perplexity indicates better performance. Perplexity is calculated as the exponent of the loss obtained from the model.
- Area under the curve (AUC): Used to evaluate the classification performance of AVP-GPT compared to an RF and an SVM model, widely used methods for antiviral activity prediction. Higher AUC signifies better classification ability.
- Confusion matrix: We used the confusion matrix to evaluate the classification performance of all models. The confusion matrix provides a detailed breakdown of true positive, true negative, false positive, and false negative predictions, allowing us to assess the model’s accuracy, sensitivity, specificity, and other relevant metrics.
- Ablation study: Conducted to assess the importance of different components and strategies within the AVP-GPT classification part.

### 2.11. AVP-GPT Fine-Tuning

Following pre-training on RSV data, AVP-GPT was fine-tuned on a dataset of INFVA and other respiratory viruses’ peptide sequences. This fine-tuning process allows the model to adapt its knowledge from RSV to the specific task of identifying antiviral peptides against INFVA and other respiratory viruses. Importantly, the AVP-GPT architecture and most hyperparameters were maintained from the pre-training process. This ensures that the core functionality of the model is preserved while allowing it to specialize in INFVA peptide analysis.

### 2.12. Software Implementation of AVP-GPT

AVP-GPT was implemented using Python 3.11.7 on a CentOS Linux 7.2 server. Deep learning functionalities were built with PyTorch 2.1.2 [16] and scikit-learn 1.4.0 [17].

### 2.13. Comparison Study

To assess the similarity between the generated AVP candidates and the reference RSV HR2/HR1 sequences, we employed the Biopython pairwise2.align.globalxx function (https://biopython.org/docs/dev/api/Bio.pairwise2.html accessed on 31 January 2024). This function calculates a global alignment score, which provides a measure of sequence similarity.

### 2.14. Phylogenetic Analysis of RSV Peptides

To explore the diversity of RSV AVPs, we performed a phylogenetic analysis. This analysis helps us understand the evolutionary relationships between different AVPs. The analysis involved the following:

- Data collection: A total of 179 anti-RSV peptide sequences, including 19 AVPs generated by AVP-GPT, 132 private AVPs from our company, and 28 public AVPs downloaded from AVPdb [10] and DRAVP [11] in May 2023.
- Sequence alignment: The MUSCLE program (v5.1) [18] was employed to align all 179 peptide sequences.
- Phylogenetic tree construction: Phylogenetic trees were built using RaxML software (v8.2.12) with the PROTGAMMAGTR model [19]. Additionally, bootstrap analysis with 1000 replicates was performed to assess the robustness of the inferred evolutionary relationships.
- Visualization and annotation: The resulting phylogenetic trees were visualized and annotated using the Interactive Tree of Life (iTOL) platform [20].
- Structure prediction: Finally, the predicted structures of the peptides were obtained using the HelixFold-Single tool [21]. This helps to understand the potential functional roles of the AVPs based on their structural features.

### 2.15. In Vitro Study

The in vitro study included the following:

- Peptide synthesis: The identified AVP candidates were synthesized using solid-phase peptide synthesis (SPPS) [22] by a commercial vendor, Chinese Peptide Company (CPC, Hangzhou, China). SPPS was chosen for its versatility and ability to produce peptides with various modifications and lengths. Crude peptides were analyzed by UPLC/MS to verify their purity and molecular weight. HPLC preparative was used to purify the peptides to high purity levels. Peptide sequencing was conducted by Shanghai Weipu Company (Shanghai, China) to confirm the correct amino acid sequence.
- Antiviral activity assay: The antiviral activity of the synthesized peptides against RSV and INFVA was evaluated by WuXi AppTec using a plaque reduction assay with the RSV strain A Long and INFV strain A/California/07/2009. HEp-2 cells were seeded in a 96-well plate overnight and infected with RSV, while MDCK cells were seeded and infected with INFVA. Peptides were serially diluted and added to the infected cells, followed by a 2 h incubation period. The medium was then replaced with fresh medium containing the same peptide concentration. Plates were incubated for 24 h at 37 °C. To quantify viral replication, the cells were fixed, permeabilized, and stained with RSV-specific or INFVA-specific antibodies and a secondary antibody. TrueBlue solution was added, and the number of plaques was counted using a microplate imaging counter.
- Cytotoxic assay: Cytotoxicity experiments were conducted in parallel with antiviral experiments to assess the potential toxicity of the peptides. HEp-2 or MDCK cells were seeded into microplates at a density of 30,000 cells per well and cultured overnight in a 5% CO_2_, 37 °C incubator. The diluted test samples were added to the cells, and a cell control (cells, no compound treatment) and a culture medium control (culture medium only, no cell or compound treatment) were set up. The final concentration of DMSO in the culture medium was 0.5%, which is a commonly used concentration that does not significantly affect cell viability. Cells were cultured for 1 day in a 5% CO_2_, 37 °C incubator. Cell viability was detected using the CCK8 assay.

## 3. Results and Discussion

### 3.1. Workflow of the AVP-GPT

The AVP-GPT workflow comprises three key stages: datasets and preprocessing, pre-training, and fine-tuning. Antiviral peptide (AVP) sequences were collected from various sources, including our group’s proprietary database (Youcare), AVPdb, and DRAVP. Data preprocessing involved removing duplicate sequences, converting EC50/IC50 values to binary labels, and ensuring data consistency. During preprocessing, antiviral peptide (AVP) sequences were fed into the AVP-GPT generation part, while both AVP and non-AVP sequences were used in the classification part. Notably, RSV data were employed for pre-training AVP-GPT, while INFVA, HPIV, SARS-CoV-2, SARS-CoV data were used for fine-tuning. This process allowed the model to adapt its knowledge to specific respiratory viruses, improving its ability to identify AVPs against these targets. The generation part of AVP-GPT generates a pool of potential peptide candidates. The classification part then evaluates each peptide candidate, predicting whether it is an AVP or a non-AVP based on its features and relationships with receptor sequences (Figure 1).

### 3.2. AVP Generation in Pre-Training

Our pre-training approach incorporates an advanced layer normalization technique. Unlike the classical method used in GPT, where layer normalization occurs after each sub-block, we adopt the GPT-2 [23] approach of placing it before each sub-block (Figure 2A). This might improve the training process by allowing the model to better learn from the incoming data at each step. During pre-training, AVP-GPT focuses on learning how to generate RSV-related antiviral peptides. We utilized a dataset of 412 non-replicated RSV peptide sequences. These sequences comprised 370 manually designed peptides curated by our team (private data) and 42 publicly available peptides (Table S1). All 370 private peptides were manually designed by our team and are not identical to any public peptides. From this collection, 160 AVPs (132 private and 28 public) were tokenized and fed into the AVP-GPT generation part (Table 1). For model training, we used a subset of 96 AVPs to balance the dataset and prevent overfitting. This decision was based on the assumption that a smaller, well-curated dataset would be sufficient for training the model to generate AVP-like sequences. 

**Figure 3:**
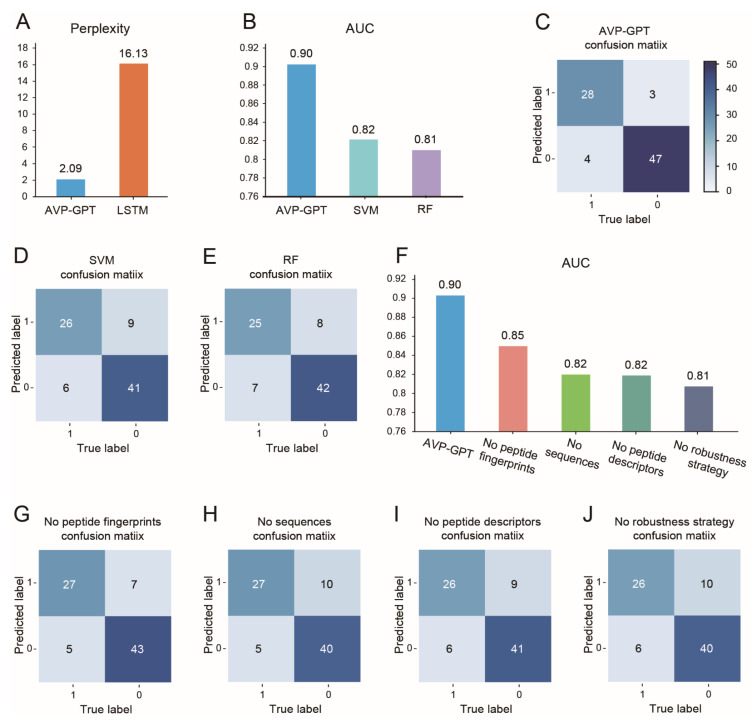
Performance comparison of AVP-GPT and ablation study results for RSV. (**A**) Perplexity comparison: Perplexity is a metric used to evaluate how well a model predicts the next element in a sequence. Lower perplexity indicates better performance. AVP-GPT achieves a remarkably lower perplexity (2.09) compared to an LSTM model (16.13). This suggests that AVP-GPT can better predict the next amino acid in an RSV peptide sequence. (**B**) AUC comparison: AUC is a metric used to assess a model’s ability to distinguish between positive and negative cases. In this context, it represents the capability to differentiate between AVPs and non-AVPs. AVP-GPT demonstrates a higher AUC (0.90) compared to an SVM model (0.82) and an RF model (0.81). This indicates that AVP-GPT performs better at classifying RSV peptides as antiviral or non-antiviral. (**C**–**E**) Confusion matrices: The confusion matrices in (**C**–**E**) demonstrate the superior performance of AVP-GPT compared to SVM and RF. AVP-GPT exhibited higher accuracy, sensitivity, and specificity, indicating its ability to accurately distinguish AVPs from non-AVPs. (**F**–**J**) Ablation study: An ablation study investigates the influence of individual components on a model’s performance. Here, it examines how removing specific parts of AVP-GPT affects its AUC and confusion matrices for RSV AVP classification. The AUC and accuracy of AVP-GPT drops when certain components are excluded: CNN model for peptide fingerprints, transformer encoder for sequence, CNN model for peptide descriptors, and method to prevent overfitting and boost robustness.

To evaluate the quality of the generated peptides, we employed perplexity, a standard language model metric. Lower perplexity signifies a more confident model with less uncertainty in its predictions [24,25]. A lower perplexity value indicates that the model is more confident in its predictions and is less likely to generate unlikely or nonsensical sequences. The AVP-GPT generation part achieved a remarkably lower perplexity (2.09) compared to a state-of-the-art generative model for peptides, LSTM (perplexity: 16.13) (Figure 3A). This result suggests that AVP-GPT can generate peptide sequences with greater confidence and potentially higher quality. Finally, AVP-GPT successfully generated 10,000 unique peptides within a single day.

### 3.3. AVP Identification in Pre-Training

During pre-training, AVP-GPT not only learns to generate peptide sequences but also refines its ability to classify them as antiviral or non-antiviral. The AVP-GPT classification part leverages a multimodal architecture consisting of transformer encoders and CNNs to process peptide sequences, peptide descriptors, and peptide fingerprints (Figure 2B–D). This multimodal approach allows the model to capture information from various data representations, potentially leading to more accurate AVP identification. Peptide sequences are processed by the transformer encoder, while peptide descriptors and fingerprints are processed by separate CNN models. The outputs from these models are then combined in fusion layers to provide a comprehensive representation of the peptide. 

The pre-training dataset was imbalanced, containing 252 non-AVPs and 160 AVPs (Table 1), reflecting the challenge of designing effective antiviral peptides. To evaluate AVP-GPT’s classification performance in this imbalanced setting, we employed the AUC metric. The AUC is insensitive to class imbalances and provides a more reliable measure of performance compared to metrics like accuracy [26,27]. An AUC of 0.5 indicates a random classifier, while 1.0 signifies perfect discrimination [28]. Generally, higher AUC values represent better classification performance. The following is a common interpretation of AUC values [29]:

- 0.6 ≤ AUC < 0.7: poor;
- 0.7 ≤ AUC < 0.8: fair;
- 0.8 ≤ AUC < 0.9: considerable;
- 0.9 ≤ AUC: excellent.

From this collection, 160 AVPs and 252 non-AVPs were tokenized and fed into the AVP-GPT generation part (Table 1), and 96 AVPs and 151 non-AVPs were used during model training.

AVP-GPT demonstrated exceptional performance in AVP identification, achieving an AUC of 0.9024, which significantly surpassed the AUC of 0.82 and 0.81 obtained by SVM and RF, respectively (Figure 3B). This indicates AVP-GPT’s superior ability to distinguish AVPs from non-AVPs.

The confusion matrices in Figure 3C–E further support these findings. AVP-GPT exhibited higher accuracy, sensitivity, and specificity compared to SVM and RF. These results highlight the effectiveness of the multimodal architecture in capturing relevant information from peptide sequences, descriptors, and fingerprints, leading to improved classification performance.

From the 10,000 unique RSV peptides generated during pre-training, the AVP-GPT classification part identified 25 peptides with a high probability (>0.9) of being AVPs. These top candidates were selected for further investigation.

### 3.4. Ablation Study in Pre-Training

Multimodal deep learning methods, which combine information from various sources, have gained traction in diverse fields due to their ability to enhance prediction accuracy [30]. By incorporating multimodal data like sequences, fingerprints, and descriptors, AVP-GPT can leverage richer information compared to models that rely on a single modality. To assess the impact of each modality on AVP-GPT’s performance, we conducted an ablation study.

Figure 3F–J summarize the ablation study results. They demonstrate that all three modalities (sequences, fingerprints, and descriptors) remarkably contribute to the model’s accuracy. When individual components were removed—the CNN model for fingerprint data, the transformer encoder for sequence data, or the CNN model for descriptor data—the AUC and the accuracy decreased, and AVP-GPT’s performance dropped below the “excellent” range in AUC. This confirms the importance of each modality in achieving optimal performance. The sequence modality captures the primary amino acid sequence information, which is crucial for understanding peptide structure and function. The fingerprint modality provides information about the overall chemical structure of the peptide, while the descriptor modality captures various physicochemical properties. By combining these modalities, AVP-GPT can leverage a more comprehensive representation of the peptides, leading to improved performance.

However, using multimodal data can also lead to overfitting, particularly with smaller datasets [30]. Our pre-training dataset consisted of only 412 samples, which could be susceptible to overfitting in a multimodal setting. To mitigate this risk and enhance the robustness of our multimodal approach, we employed a strategy involving the random deletion of 50% of the receptor sequences, peptide fingerprints, and peptide descriptors. These deleted values were then padded with “<PAD>” or zeros. As shown in Figure 3F–J, the strategy proved to be effective in preventing overfitting and improving the model’s overall accuracy. The AUC score and the accuracy dropped obviously when this strategy was not implemented, highlighting its importance in achieving reliable results. While this strategy was effective in our case, it is important to consider other potential overfitting mitigation techniques, such as regularization or data augmentation, which may be suitable for different datasets or model architectures.

### 3.5. AVP-GPT Fine-Tuning Study

Large pre-trained models like GPT have revolutionized the field of artificial intelligence by demonstrating exceptional capabilities. Their extensive knowledge gained during pre-training allows them to be fine-tuned for specific tasks with minimal additional data [31]. 

**Figure 4:**
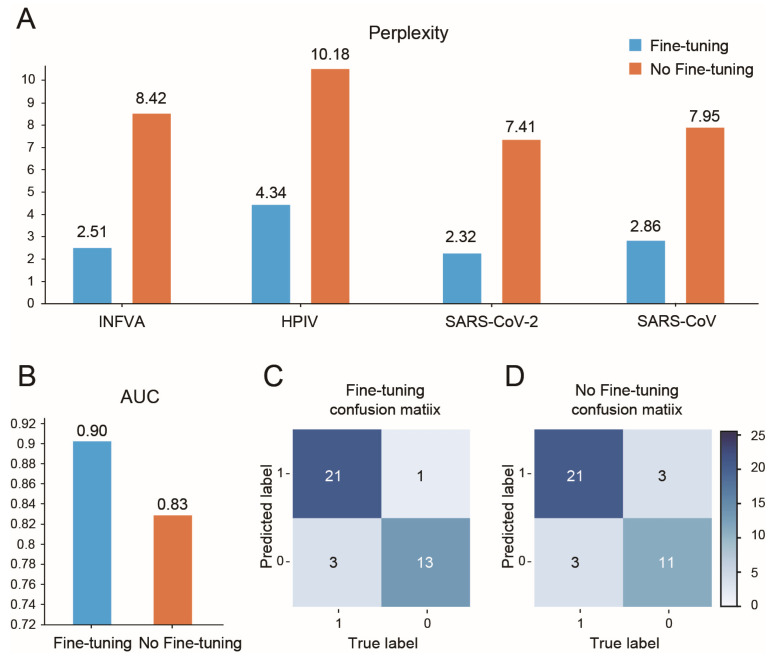
Impact of AVP-GPT fine-tuning on INFVA, SARS-CoV, SARS-CoV-2, and HPIV performance. (**A**) Perplexity: The fine-tuned AVP-GPT model achieved lower perplexity values for all four viral targets (INFVA, SARS-CoV, SARS-CoV-2, and HPIV) compared to the non-fine-tuned model. This indicates that fine-tuning improved the model’s ability to predict the next amino acid in peptide sequences from these viruses. (**B**) AUC: the fine-tuned AVP-GPT also demonstrated higher AUC scores for all four viral targets, indicating its improved ability to classify peptides from these viruses as antiviral or non-antiviral. (**C**,**D**) Confusion matrices: the confusion matrices in (**C**,**D**) further illustrate the improved performance of the fine-tuned AVP-GPT model.

In our case, the limited dataset of INFVA, HPIV, SARS-CoV-2, and SARS-CoV peptides (192 total) (Table S2) presented a challenge. To address this, we leveraged the RSV pre-trained AVP-GPT and fine-tuned it on the INFVA, HPIV, SARS-CoV-2, and SARS-CoV dataset. Fine-tuning remarkably improved AVP-GPT’s performance in these new tasks compared to not using fine-tuning or training a new model from scratch (Figure 4). In total, 120 AVPs and 72 non-AVPs were tokenized and fed into the AVP-GPT classification part, and 72 AVPs and 43 non-AVPs were used during the model training process.

The fine-tuned model achieved a lower perplexity (2.32–4.34), a higher AUC (0.90), and a higher accuracy compared to the non-fine-tuned model (perplexity: 7.41–10.18, AUC: 0.83). These results demonstrate the effectiveness of fine-tuning AVP-GPT for downstream applications with a new virus target, showcasing its versatility; moreover, AVP-GPT can potentially identify antiviral peptides for different viruses despite training on a specific dataset initially. From the ten thousand unique INFVA peptides generated during pre-training, the AVP-GPT classification part identified five peptides with a high probability (>0.9) of being AVPs.

### 3.6. Comparison Study and Phylogenetic Analysis

It is well established that peptides derived from the HR domain of the RSV F protein can exhibit antiviral activity. For instance, the peptide T118, derived from the HR2 region, demonstrates potent anti-RSV activity with an EC50 value of 0.05 μM [32]. Similar peptides can competitively bind to the HR1 region, blocking the natural HR2-HR1 interaction and inhibiting the RSV fusion process.

**Figure 5:**
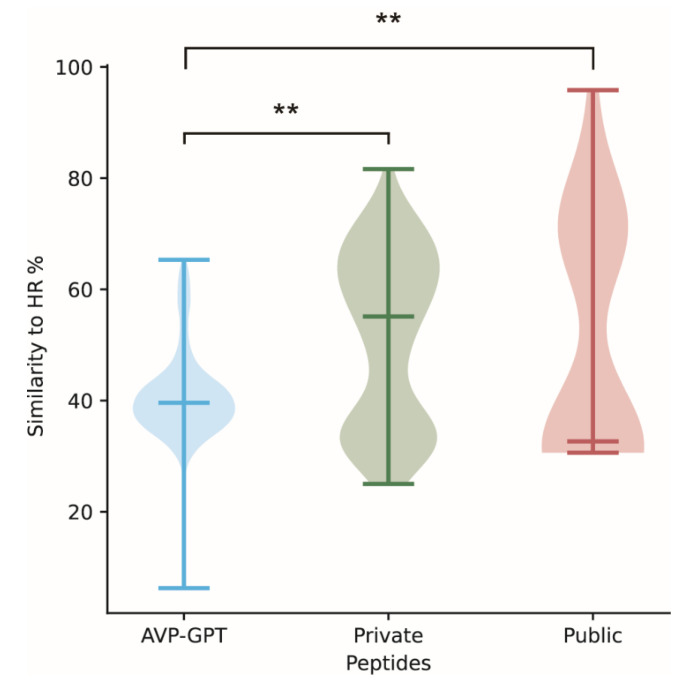
Comparison of peptide similarity to RSV reference sequences (HRs). This figure compares the similarity of three groups of peptides to reference sequences from RSV-HR1 and HR2. The following is a breakdown of the compared groups: 10,000 AVP-GPT-designed peptides, 370 private peptides, and 42 public peptides. For each peptide, the maximum similarity score between HR1 and HR2 was chosen. **: A statistical test (Wilcoxon test) was performed with a *p*-value less than 2.2 × 10−16, indicating a highly significant difference in similarity between the groups.

We compared the similarity of the 10,000 AVP-GPT-generated RSV peptides to known HR sequences. As expected for novel candidates, the AVP-GPT peptides displayed highly significant lower sequence similarity to the HR region compared to manually designed RSV peptides and publicly available peptides (Table 2, Figure 5). This suggests a higher degree of mutation and potentially novel functionalities within the AVP-GPT-generated pool. Interestingly, 8644 peptides (86%) from the 10,000-peptide pool possessed the characteristic α-helix structure, which is the native conformation of the HR2 domain. This percentage was higher compared to the α-helix content observed in the public and private RSV peptide datasets (Table 2). This finding suggests that a remarkable portion of the AVP-GPT-generated peptides retain the crucial structural feature associated with antiviral activity.

**Figure 6:**
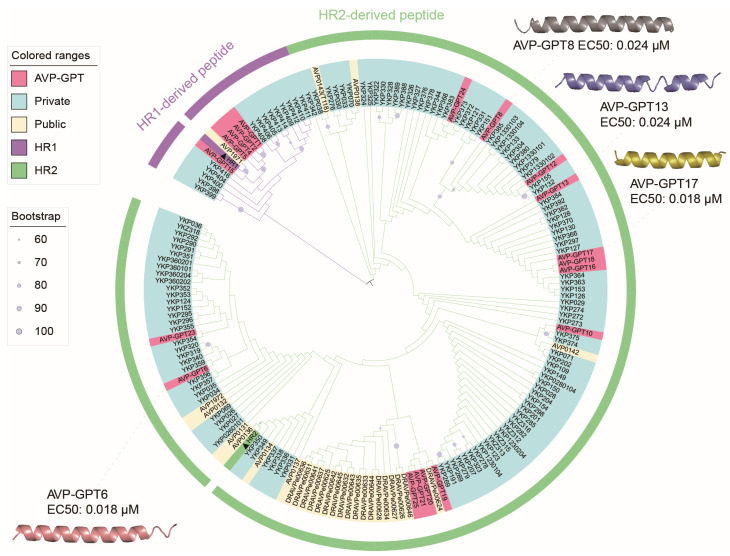
Phylogenetic tree of anti-RSV peptides. Nineteen peptides designed by AVP-GPT, one hundred thirty-two private peptides, twenty-eight public peptides, RSV HR1, and RSV HR2 are highlighted in Indian red, pale turquoise, floral white, medium orchid, and pale green, respectively. RSV HR1 and RSV HR2 are labeled with black triangles. Four peptides (AVP-GPT6, AVP-GPT8, AVP-GPT13, and AVP-GPT17) out of the nineteen anti-RSV peptides were shown to be good candidates for anti-RSV, with EC50 values around 0.02 uM.

Furthermore, phylogenetic analysis revealed that the 19 AVPs identified from the 10,000 peptides contribute to the overall diversity of antiviral candidates (Figure 6). Notably, five AVPs originated from the HR1 region, while fourteen stemmed from the HR2 region. This highlights that peptides derived from both HR1 and HR2 can exhibit anti-RSV activity, although AVP-GPT identified a higher number of HR2-derived candidates. Targeting HR1, located near the fusion peptide sequence, allows these peptides to potentially disrupt the steps required for RSV entry into the host cell.

### 3.7. Result of In Vitro Study

Encouragingly, 19 out of the 25 identified AVP candidates against RSV exhibited antiviral activity in vitro, with EC50 values below 10 μM (Table 3, Figure 6). Notably, four particularly potent peptides, all derived from the HR2 region and boasting EC50 values of around 0.02 uM, were discovered by AVP-GPT. This demonstrates the model’s ability to generate highly effective antiviral candidates. Compared to both our private manually designed peptides and publicly available peptides, the AVP-GPT-generated candidates displayed a remarkably higher antiviral success rate (Table 4). Additionally, three peptides against INFVA exhibited EC50 values below 4 μM, demonstrating their promising antiviral activity against this virus (Table 5). This highlights the superiority of AVP-GPT over conventional design methods. The low cytotoxicity of the identified AVPs is another promising aspect. All twenty-five candidates against RSV and five candidates against INFVA exhibited CC50 (cytotoxic concentration) values exceeding 1 μM, indicating a good safety profile. These findings collectively suggest that the four AVPs designed by AVP-GPT represent attractive candidates for further development as anti-RSV and anti-INFVA therapeutics.

## 4. Conclusions

In this study, we introduce AVP-GPT, a novel end-to-end transformer-based deep learning model for antiviral peptide design. AVP-GPT demonstrates exceptional capabilities in both RSV and INFV applications. It has the remarkable capacity to generate 10,000 unique peptides and achieves high accuracy in identifying potential AVPs within two days. These AVP-GPT-generated peptides exhibit highly significant lower sequence similarity to the RSV HR region compared to existing RSV peptides, suggesting a high degree of mutation and potentially novel functionalities. Phylogenetic analysis confirms the substantial diversity of the generated AVPs. Furthermore, in vitro experiments validate the efficacy of AVP-GPT, with four identified peptides demonstrating potent anti-RSV activity and three peptides anti-INFV activity. These findings highlight AVP-GPT’s potential as a powerful tool for accelerating the discovery and development of novel antiviral therapeutics. While this study provides promising results, further validation and optimization are necessary to fully realize the potential of AVP-GPT in drug development.

## Figures and Tables

**Table 1:** Dataset summary of AVP-GPT generation and classification parts of pre-training.

Data Category	Generation Part (AVP Sequence Creation)	Classification Part (AVP Identification)
Private AVPs	132	132
Private non-AVPs	Not Available (NA)	238
Public AVPs	28	28
Public non-AVPs	Not Available (NA)	14
Total	160	412

Note: “NA” stands for “Not Available”. This indicates that non-AVP data were not used during the generation of new peptide sequences.

**Table 2:** Comparison of anti-RSV peptide properties of AVP-GPT, private, and public peptides.

Category	Similarity to HR (%)	Alpha-Helical Content (%)	Beta-Sheet Content (%)
AVP-GPT peptides	6.25–65.31	86.44	0.28
Private peptides	25.00–81.63	82.16	0.74
Public peptides	30.61–95.83	45.24	0.00

Note: similarity to HR: choose the maximum similarity score between HR1 and HR2.

**Table 3:** Result of in vitro study for 25 AVP-GPT peptides targeting the RSV.

ID	EC50 (μM)	CC50 (μM)
AVP-GPT1	1.281	>8
AVP-GPT2	0.418	>8
AVP-GPT3	>8	>8
AVP-GPT4	1.246	>8
AVP-GPT5	0.698	>8
AVP-GPT6	0.018	4.907
AVP-GPT7	>8	>8
AVP-GPT8	0.024	1.074
AVP-GPT9	>8	>8
AVP-GPT10	0.4522	>8
AVP-GPT11	>8	>8
AVP-GPT12	6.649	5.813
AVP-GPT13	0.024	4.257
AVP-GPT14	>2	4.167
AVP-GPT15	5.472	3.372
AVP-GPT16	0.096	>8
AVP-GPT17	0.018	1.428
AVP-GPT18	0.099	>8
AVP-GPT19	2.219	>8
AVP-GPT20	0.296	>8
AVP-GPT21	5.037	>8
AVP-GPT22	>2	1.519
AVP-GPT23	5.529	5.138
AVP-GPT24	6.823	>8
AVP-GPT25	5.226	4.022

**Table 4:** Antiviral rate comparison of AVP-GPT, private, and public anti-RSV peptides.

Category	Low Antiviral Rate (EC50/IC50 < 10 uM, %)	High Antiviral Rate (EC50/IC50 < 0.03 uM, %)
AVP-GPT peptides	76.00	16.00
Private peptides	35.68	5.41
Public peptides	66.67	0.00

**Table 5:** Result of in vitro study for 5 AVP-GPT peptides targeting the INFVA.

ID	EC50 (μM)	CC50 (μM)
AVP-GPT26	13.30	9.29
AVP-GPT27	2.36	>40
AVP-GPT28	1.27	>40
AVP-GPT29	11.19	>40
AVP-GPT30	3.98	>40

## Data Availability

To facilitate access to the private dataset, interested researchers can contact us at songgengshen@youcareyk.com or zhaohuajian@youcareyk.com. Upon request, we will provide the dataset subject to appropriate agreements and permissions. Once the patent is granted, we plan to explore the possibility of depositing the dataset and model weights in a public repository to enhance accessibility and reproducibility.

## Supplementary Materials

The following supporting information can be downloaded at https://www.mdpi.com/article/10.3390/v16111673/s1. Table S1. Publicly available RSV dataset used for AVP-GPT pre-training. Table S2. Publicly available INFVA and other respiratory virus dataset used for AVP-GPT fine-tuning.

## References

[1] Tripathi N.M., Bandyopadhyay A. (2022). High throughput virtual screening (HTVS) of peptide library: Technological advancement in ligand discovery. Eur. J. Med. Chem..

[2] Sun Z., Pan Y., Jiang S., Lu L. (2013). Respiratory syncytial virus entry inhibitors targeting the F protein. Viruses.

[3] López-Martínez R., Ramírez-Salinas G.L., Correa-Basurto J., Barrón B.L. (2013). Inhibition of influenza A virus infection in vitro by peptides designed in silico. PLoS ONE.

[4] Muller A.T., Hiss J.A., Schneider G. (2018). Recurrent neural network model for constructive peptide design. J. Chem. Inf. Model..

[5] Ali F., Kumar H., Alghamdi W., Kateb F.A., Alarfaj F.K. (2023). Recent advances in machine learning-based models for prediction of antiviral peptides. Arch. Comput. Methods Eng..

[6] Pang Y., Yao L., Jhong J.H., Wang Z., Lee T.Y. (2021). AVPIden: A new scheme for identification and functional prediction of antiviral peptides based on machine learning approaches. Brief. Bioinform..

[7] Vaswani A., Shazeer N., Parmar N., Uszkoreit J., Jones L., Gomez A.N., Kaiser L., Polosukhin I. Attention is all you need. Proceedings of the 31st Conference on Neural Information Processing Systems (NIPS 2017).

[8] Devlin J., Chang M.W., Lee K. (2018). Bert: Pre-training of deep bidirectional transformers for language understanding. arXiv.

[9] Radford A., Narasimhan K., Salimans T. (2018). Improving Language Understanding by Generative Pre-Training. https://www.mikecaptain.com/resources/pdf/GPT-1.pdf.

[10] Qureshi A., Thakur N., Tandon H., Kumar M. (2014). AVPdb: A database of experimentally validated antiviral peptides targeting medically important viruses. Nucleic Acids Res..

[11] Liu Y., Zhu Y., Sun X., Ma T., Lao X., Zheng H. (2023). DRAVP: A comprehensive database of antiviral peptides and proteins. Viruses.

[12] Ji Y., Zhou Z., Liu H., Davuluri R.V. (2021). DNABERT: Pre-trained Bidirectional Encoder Representations from Transformers model for DNA-language in genome. Bioinformatics.

[13] Singh M., Berger B., Kim P.S. (1999). LearnCoil-VMF: Computational evidence for coiled-coil-like motifs in many viral membrane-fusion proteins. J. Mol. Biol..

[14] Chou K.C. (2001). Prediction of protein cellular attributes using pseudo-amino acid composition. Proteins Struct. Funct. Bioinform..

[15] Dozat T. (2016). Incorporating Nesterov Momentum into Adam. https://openreview.net/forum?id=OM0jvwB8jIp57ZJjtNEZ.

[16] Paszke A., Gross S., Chintala S., Chanan G., Yang E., DeVito Z., Lin Z., Desmaison A., Antiga L., Lerer A. (2017). Automatic differentiation in pytorch. Openreview.

[17] Pedregosa F., Varoquaux G., Gramfort A., Michel V., Thirion B., Grisel O., Blondel M., Prettenhofer P., Weiss R., Dubourg V. (2011). Scikit-learn: Machine learning in Python. J. Mach. Learn. Res..

[18] Edgar R.C. (2022). Muscle5: High-accuracy alignment ensembles enable unbiased assessments of sequence homology and phylogeny. Nat. Commun..

[19] Stamatakis A. (2014). RAxML version 8: A tool for phylogenetic analysis and post-analysis of large phylogenies. Bioinformatics.

[20] Letunic I., Bork P. (2021). Interactive Tree Of Life (iTOL) v5: An online tool for phylogenetic tree display and annotation. Nucleic Acids Res..

[21] Fang X., Wang F., Liu L., He J., Lin D., Xiang Y., Zhu K., Zhang X., Wu H., Li H. (2023). A method for multiple-sequence-alignment-free protein structure prediction using a protein language model. Nat. Mach. Intell..

[22] Gaillard V., Galloux M., Garcin D., Eléouët J.-F., Le Goffic R., Larcher T., Rameix-Welti M.-A., Boukadiri A., Héritier J., Segura J.-M. (2017). A short double-stapled peptide inhibits respiratory syncytial virus entry and spreading. Antimicrob. Agents Chemother..

[23] Radford A., Wu J., Child R., Luan D., Amodei D., Sutskever I. (2019). Language models are unsupervised multitask learners. OpenAI Blog.

[24] Hao Y., Mendelsohn S., Sterneck R., Martinez R., Frank R. (2020). Probabilistic predictions of people perusing: Evaluating metrics of language model performance for psycholinguistic modeling. arXiv.

[25] Moret M., Grisoni F., Katzberger P., Schneider G. (2022). Perplexity-based molecule ranking and bias estimation of chemical language models. J. Chem. Inf. Model..

[26] Sulam J., Ben-Ari R., Kisilev P. Maximizing AUC with Deep Learning for Classification of Imbalanced Mammogram Datasets. Proceedings of the Eurographics Workshop on Visual Computing for Biology and Medicine (VCBM).

[27] Liu M., Yuan Z., Ying Y., Yang T. (2019). Stochastic auc maximization with deep neural networks. arXiv.

[28] Carrington A.M., Manuel D.G., Fieguth P.W., Ramsay T., Osmani V., Wernly B., Bennett C., Hawken S., McInnes M., Magwood O. (2021). Deep ROC analysis and AUC as balanced average accuracy to improve model selection, understanding and interpretation. arXiv.

[29] Çorbacıoğlu Ş.K., Aksel G. (2023). Receiver operating characteristic curve analysis in diagnostic accuracy studies: A guide to interpreting the area under the curve value. Turk. J. Emerg. Med..

[30] Stahlschmidt S.R., Ulfenborg B., Synnergren J. (2022). Multimodal deep learning for biomedical data fusion: A review. Brief. Bioinform..

[31] Han X., Zhang Z., Ding N., Gu Y., Liu X., Huo Y., Qiu J., Yao Y., Zhang A., Zhang L. (2021). Pre-trained models: Past, present and future. AI Open.

[32] Lambert D.M., Barney S., Lambert A.L., Guthrie K., Medinas R., E Davis D., Bucy T., Erickson J., Merutka G., Petteway S.R. (1996). Peptides from conserved regions of paramyxovirus fusion (F) proteins are potent inhibitors of viral fusion. Proc. Natl. Acad. Sci. USA.

